# The Heteromultimeric Debranching Enzyme Involved in Starch Synthesis in Arabidopsis Requires Both Isoamylase1 and Isoamylase2 Subunits for Complex Stability and Activity

**DOI:** 10.1371/journal.pone.0075223

**Published:** 2013-09-30

**Authors:** Maria Sundberg, Barbara Pfister, Daniel Fulton, Sylvain Bischof, Thierry Delatte, Simona Eicke, Michaela Stettler, Steven M. Smith, Sebastian Streb, Samuel C. Zeeman

**Affiliations:** 1 Department of Biology, ETH Zurich, Zurich, Switzerland; 2 Institute of Molecular Plant Sciences, University of Edinburgh, Edinburgh, Scotland, United Kingdom; 3 School of Chemistry and Biochemistry, University of Western Australia, Crawley, Western Australia, Australia; Umeå Plant Science Centre, Sweden

## Abstract

Isoamylase-type debranching enzymes (ISAs) play an important role in determining starch structure. Amylopectin – a branched polymer of glucose – is the major component of starch granules and its architecture underlies the semi-crystalline nature of starch. Mutants of several species lacking the ISA1-subclass of isoamylase are impaired in amylopectin synthesis. Consequently, starch levels are decreased and an aberrant soluble glucan (phytoglycogen) with altered branch lengths and branching pattern accumulates. Here we use TAP (tandem affinity purification) tagging to provide direct evidence in Arabidopsis that ISA1 interacts with its homolog ISA2. No evidence for interaction with other starch biosynthetic enzymes was found. Analysis of the single mutants shows that each protein is destabilised in the absence of the other. Co-expression of both ISA1 and ISA2 *Escherichia coli* allowed the formation of the active recombinant enzyme and we show using site-directed mutagenesis that ISA1 is the catalytic subunit. The presence of the active isoamylase alters glycogen biosynthesis in *E. coli*, resulting in colonies that stain more starch-like with iodine. However, analysis of the glucans reveals that rather than producing an amylopectin like substance, cells expressing the active isoamylase still accumulate small amounts of glycogen together with a population of linear oligosaccharides that stain strongly with iodine. We conclude that for isoamylase to promote amylopectin synthesis it needs to act on a specific precursor (pre-amylopectin) generated by the combined actions of plant starch synthase and branching enzyme isoforms and when presented with an unsuitable substrate (i.e. *E. coli* glycogen) it simply degrades it.

## Introduction

Starch is the major storage carbohydrate in plants. It is important for mankind as a source of nutrition but also as a renewable industrial raw material. Amylopectin is the major constituent of starch. It is a huge, branched polymer, containing between 10^5^ and 10^6^ glucose residues. In amylopectin, linear chains with an average length of 20–25 α-1,4-linked residues are connected to each other by α-1,6-branch points, which account for 4–5% of the linkages. The combination of chain length distribution and branching pattern results in a racemose or tree-like architecture. This architecture enables clusters of unbranched chain segments within the amylopectin molecules to form secondary and tertiary structures, resulting in a semi-crystalline matrix and conferring the insoluble nature of starch [1]. Glycogen is the comparable storage polymer found in archaea, bacteria, and many eukaryotes. Like amylopectin, glycogen is composed of α-1,4 linked glucose chains that are branched through α-1,6 linkages. However, the α-1,6 branches account for 7–10% of the linkages and are evenly distributed within the glycogen particle. This is thought to prevent the formation of secondary and tertiary structures, rendering glycogen soluble.

Amylopectin synthesis involves 1) the formation of the glucosyl donor, ADP-glucose, 2) glucan chain elongation by the glucosyl transferase reaction of starch synthases, 3) α-1,6-branch point formation by the glucanotransferase reaction of branching enzymes and 4) a degree of α-1,6-branch point hydrolysis by debranching enzymes. While glycogen synthesis typically requires only a single isoform of glycogen synthase and a single isoform of branching enzyme, amylopectin biosynthesis requires multiple isoforms of these enzymes. The isoforms have distinct properties and each contributes in a slightly different way to determining amylopectin architecture (e.g. by preferentially elongating or transferring chains of a certain length). However, the loss of a specific debranching enzyme (DBE) activity also leads to a striking alteration in glucan structure causing the accumulation of a soluble glucan, called phytoglycogen, instead of or as well as insoluble starch granules. Phytoglycogen has both an altered distribution of chain lengths and an altered distribution of branch points, compared with amylopectin. This phenotype has been observed in various plant tissues including the developing endosperms of barley [2], maize [3] and rice [4], the green algae *Chlamydomonas reinhardtii* [5]; Arabidopsis leaves [6] and potato tubers [7]. The phytoglycogen-accumulating phenotype is explained with the idea that debranching removes wrongly-positioned branch points that impede or delay the formation of the semi-crystalline amylopectin structures. However, the way in which debranching enzymes recognise wrongly-positioned branch points is not fully understood.

Four genes encoding DBE proteins are conserved in plants [8]: three isoamylase type (EC: 3.2.1.68, designated ISA1, ISA2 and ISA3) and one limit dextrinase type (EC: 3.2.1.142, designated LDA). Isoamylases and limit-dextrinase have different substrate specificities. For example, LDA prefers the yeast glucan pullulan to amylopectin or glycogen, and is often called pullulanase for this reason [9]. The ISA1 class of DBE is the one primarily associated with amylopectin synthesis and its loss causes phytoglycogen accumulation in all species studied so far. In the dicots Arabidopsis and potato, loss of ISA2 causes the same phenotype as the loss of ISA1 and there is good evidence that the two proteins form a heteromultimeric enzyme [7], [10]–[12]. Furthermore, protein sequence analysis suggests that the ISA2 subunit is not catalytically active. This is due to the substitution of multiple amino acid residues shown to be required for catalysis in other members of the α-amylase superfamily [13]. Interestingly, when constructs for antisense suppression of *ISA1* or *ISA2* were transformed into potato, the expression of both genes was suppressed [7]. In Arabidopsis, mutation of *ISA2* also caused the reduction of ISA1 protein [10]. These data suggest that the expression of the genes may be coordinated and/or that the complex stability may be dependent on the presence of both proteins. The situation differs in the members of the Poaceae. In the starchy endosperm of both maize and rice, ISA1 is predominantly present and active as a homomultimer, with only a small fraction forming a heteromultimer with ISA2 [14], [15]. In both cases, loss of ISA2 does not affect the expression of the ISA1 protein and does not result in phytoglycogen accumulation [16], [17]. Mutant analysis in maize suggests that either the homomultimer or the heteromultimer can promote starch synthesis [16]. However, in rice, it is intriguing that over-expression of ISA2 promotes the formation of the ISA1–ISA2 heteromultimer and impairs starch synthesis, suggesting that the homomultimer is critical [17].

Loss of the other DBEs, LDA and ISA3 does not result in phytoglycogen accumulation [11], [18], although when combined with mutations in *ISA1* and *ISA2*, the phytoglycogen-accumulating phenotype in Arabidopsis is enhanced [19]. LDA and ISA3 have their highest activities on β-limit dextrins - amylopectin in which the outer chains have been digested with the exo-acting β-amylase [9], [12], [13]. This probably resembles the substrate of the enzymes *in vivo*, as they are responsible for the debranching that occurs during starch degradation.

Here, we further investigated the functions of the ISA1/ISA2 enzyme in Arabidopsis both *in vivo* and *in vitro*. We demonstrate through affinity tagging that the two proteins do indeed form a complex, that the subunits are interdependent for their stability, and that ISA1 is the catalytic subunit. Furthermore, we provide a first indication of the structure of the enzyme multimer and, through expression of the complex in *E. coli*, show that it is capable of modifying glucan structure.

## Results

### Further Evidence for the ISA1–ISA2 Complex

Previous experiments provided indirect evidence that ISA1 and ISA2 form a complex in Arabidopsis. First, mutation of either gene abolishes the activity and results in the same phenotype and second, using native-PAGE methods, Delatte et al. [10] observed that the native enzyme has a high molecular weight (the activity co-eluted with ribulose-1,5-bisphophate carboxylase/oxygenase (Rubisco), known to have a native mass of around 500 kD). We performed a series of experiments to provide direct evidence for the complex.

**Figure 1:**
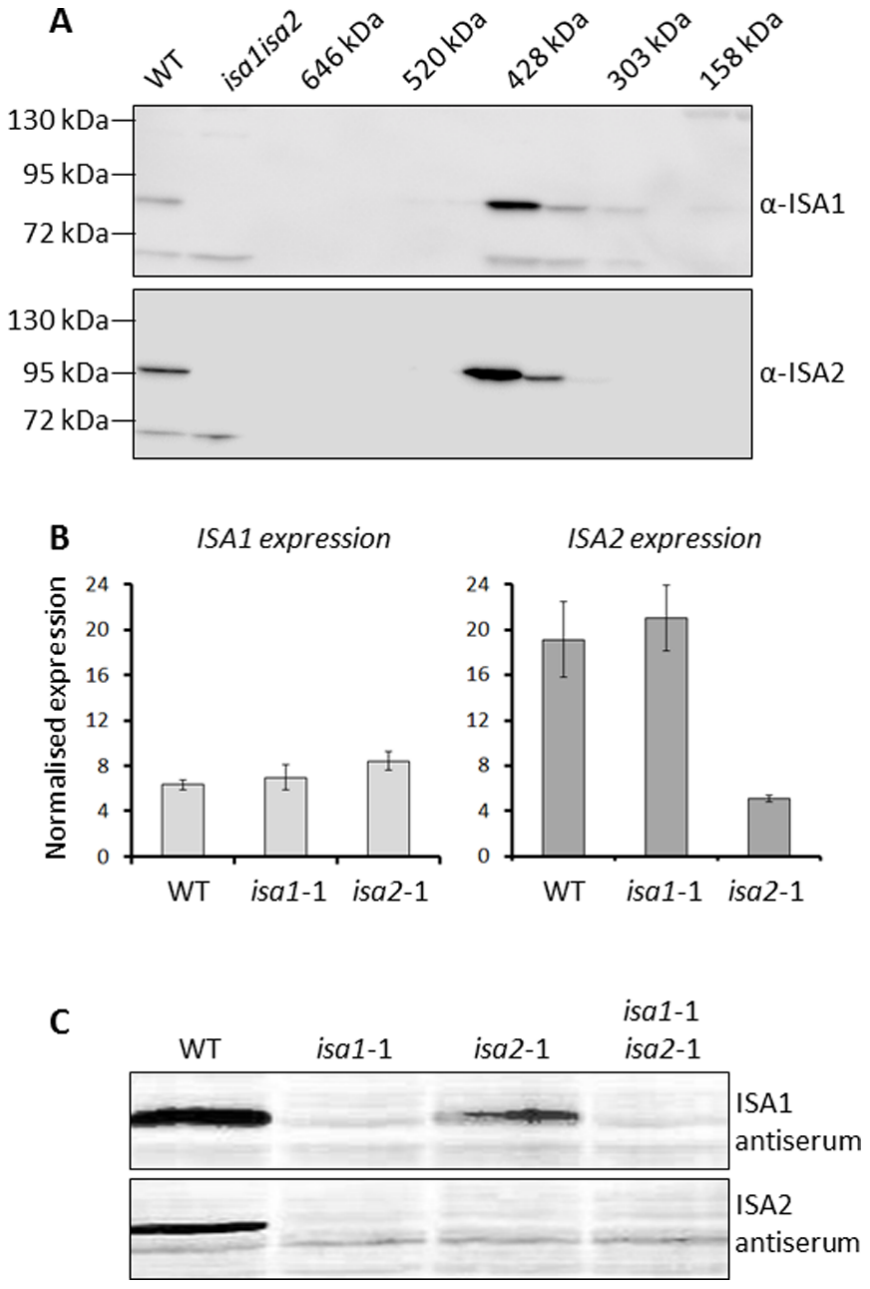
ISA1 and ISA2 co-elutes in a high molecular weight complex. A. Gel filtration chromatography of extracts of wild-type Arabidopsis leaves. Fractions from a Sephacryl HiLoad 200 prep grade column were collected, concentrated and separated by SDS-PAGE. ISA1 and ISA2 were detected by immunoblot analysis probed with specific antibodies. For comparison crude extracts of the wild type and the *isa1isa2* double mutants are in the left two lanes. Molecular masses based on the elution positions of known standards are indicated. B. RNA was extracted from 20-day old plants harvested at the end of day, reverse-transcribed to cDNA and transcript levels were analysed by real-time PCR. The expression of *ISA1* and *ISA2* in wild-type and the different mutants are shown relative to that of the housekeeping gene *PP2A*. C. Soluble proteins in extracts of wild-type and *isa* mutant Arabidopsis leaves were analysed by SDS-PAGE followed by immunoblotting with specific antibodies. 50 µg of total protein was loaded into each lane.

Using a calibrated Sephacryl column for gel filtration chromatography, we separated proteins in crude extracts of wild-type leaves according to their native molecular weight. The eluted proteins were collected in fractions and analysed by SDS-PAGE and immunoblotting with antibodies raised against peptide sequences specific to either ISA1 or ISA2 [10] (see also Materials and Methods). Both ISA1 and ISA2 proteins co-eluted in fractions corresponding to the 428 kDa marker (Fig. 1A). Next, we investigated how the loss of ISA1 affects the gene expression and protein abundance of ISA2 and *vice versa*. Microarray studies revealed that *ISA1* and *ISA2* expression is highest at the end of the day [20]. Therefore, we harvested leaves of the wild type and the mutant lines *isa1*-1 (hereafter referred to as *isa1*) and *isa2*-1 (hereafter referred to as *isa2*) at this time point and prepared cDNA from extracted mRNA. Quantitative RT-PCR revealed that *ISA1* expression was similar to the wild type in *isa1*-1 (Fig. 1B). This was not unexpected, as the primers used for RT-PCR amplify a sequence upstream of the insertion site of the T-DNA that disrupts the gene [10]. Thus, expression from the *ISA1* promoter appears to be unchanged. In *isa2*, *ISA1* expression was also unchanged. *ISA2* expression was significantly decreased in *isa2*, suggesting that the non-sense mutation decreases mRNA stability. However, *ISA2* expression was unaltered in *isa1*. Therefore, there is no evidence that the transcription of the two genes is linked in Arabidopsis, as was suggested for potato [7].

We extracted soluble proteins from the leaves of the wild type and the mutants and probed SDS-PAGE gel blots with the specific antibodies. As previously reported, ISA1 was missing in *isa1* and reduced in abundance in *isa2* (Fig. 1C). Interestingly, our analysis also revealed that ISA2 was undetectable both *isa2* and *isa1* mutants. Thus, in each *isa* mutant, the gene encoding the other *ISA* gene is expressed, but the protein fails to accumulate to normal levels. This observation is consistent with the idea that the proteins form a complex and that each subunit (particularly ISA2) is less stable in the absence of its partner.

### Tandem-Affinity-Purification (TAP)-tagging of ISA2

**Figure 2:**
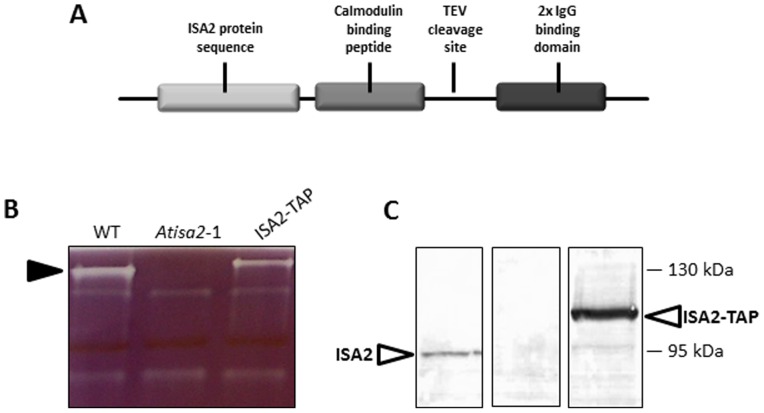
TAP-tagging ISA2 restores the isoamylase activity. A. Schematic overview of the structure of the TAP-tag fused to the *ISA2* coding sequence (not to scale). B. The *ISA2-TAP*-expressing plants were analysed for isoamylase activity on amylopectin-containing native gels. C. Immunoblot analysis of SDS-PAGE gels, probed with antiserum raised against and ISA2-specific peptide. The TAP-tagged ISA2 is 20 kDa larger than native ISA2, corresponding to the predicted molecular weight of the tag.

To confirm the interaction between ISA1 and ISA2 and to identify further potential interaction partners, a tandem-affinity-purification (TAP) approach was employed [21], [22]. The coding region of ISA2 was fused to a sequence encoding a C-terminal TAP-tag (Fig. 2A) and the construct was expressed in the *isa2* mutant line under the 35S CaMV promoter.

We used native PAGE in amylopectin-containing gels to check if the isoamylase activity missing in the *isa2* mutant was restored in *ISA2-TAP* transformed plants. Indeed, iodine staining of the gels revealed a band of activity at the top of the gel for the wild type, which was missing in the *isa2* mutant but present in the *ISA2-TAP* lines (Fig. 2B). The activity in the *ISA2-TAP* extract showed slightly lower mobility on the gel, presumably caused by presence of the ∼20 kDa tag on the ISA2 subunits. This was also visible by SDS-PAGE followed by immunoblotting (Fig. 2C). The native molecular weight of the isoamylase activity found in ISA2-TAP transformed plants was analysed by gel filtration chromatography followed by immunoblotting. Both ISA1 and ISA2-TAP co-eluted in a high molecular weight complex of circa 530 kDa compared to 428 kDa in wild-type plants (not shown).

**Figure 3:**
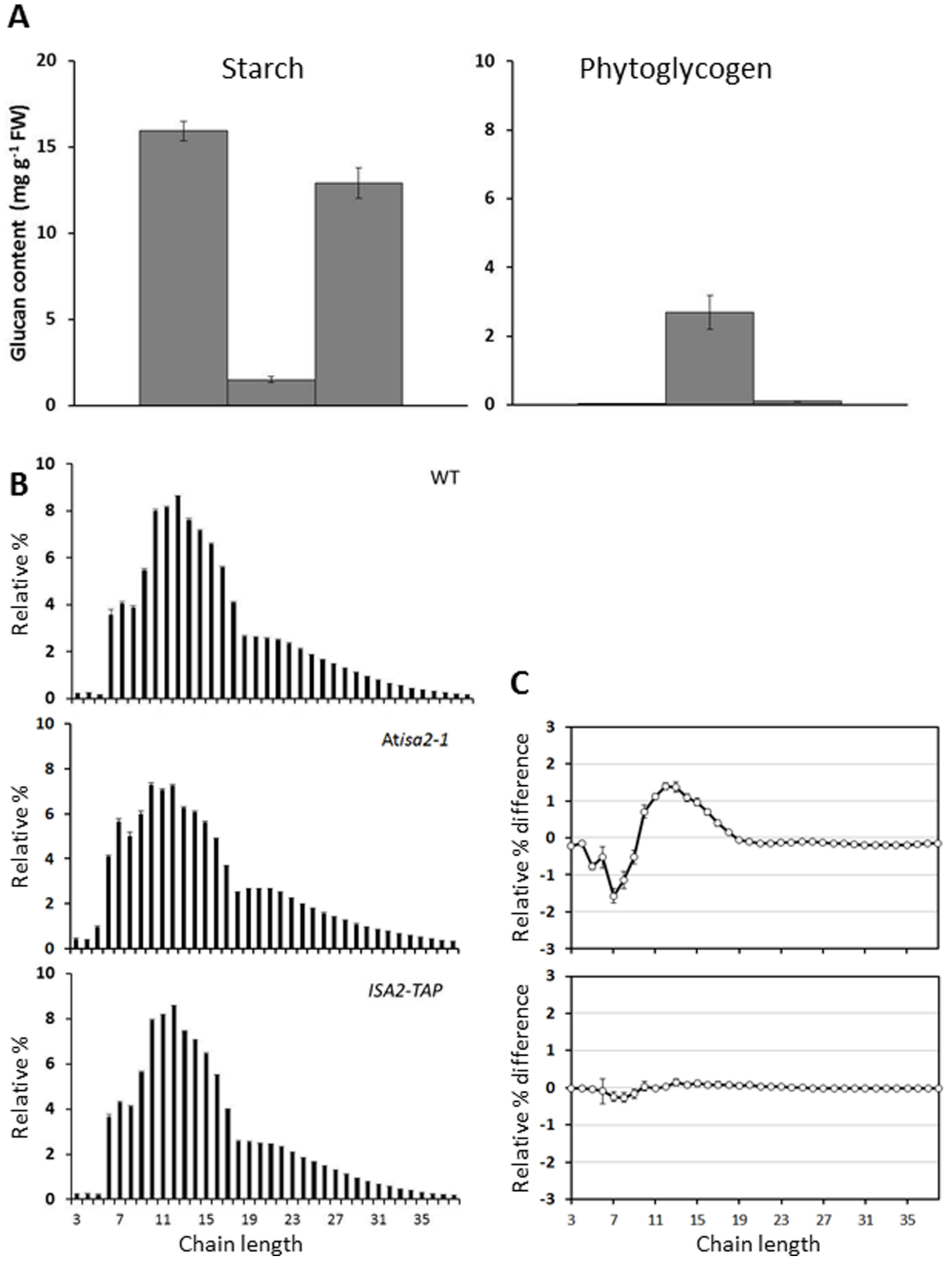
TAP-tagged ISA2 complements the *isa2*-1 phenotype. Leaves from individual plants of wild-type, *isa2*-1 mutant and *ISA2-TAP* lines were harvested at the end of the day and immediately frozen in liquid N_2_. The insoluble and soluble glucans were extracted using perchloric acid. A. Starch and phytoglycogen accumulation was measured after enzymatic hydrolysis to glucose. Each value is the mean ± SE of five biological replicates. B. The chain-length distribution (CLD) of insoluble glucans was analysed with HPEAC-PAD. Peak areas were summed and the areas of individual peaks were calculated as a percentage of the total. Equal amounts of glucan from the insoluble material from biological replicates in (A) were pooled. The means ± SE of four technical replicate digests are shown. C. Difference plot derived by subtracting the relative percentage values of wild-type (WT) amylopectin from the *ISA2-TAP* line and *isa2*-1 mutant.

We investigated whether the activity restored by transformation with *ISA2-TAP* was functional in starch biosynthesis by analysing the phenotype of the transformed lines. The *isa2* mutant accumulated both starch and phytoglycogen, as previously reported [6], [10](Fig. 3A). Starch levels were restored in *ISA2-TAP* plants almost to the level seen in wild-type plants (Fig. 3A). There was no measurable phytoglycogen in the *ISA2-TAP* plants. Structural analyses using high performance anion exchange chromatography with pulsed amperometric detection (HPAEC-PAD) revealed that the starch synthesised in the *ISA2-TAP* plants had an amylopectin chain-length distribution similar to the wild type and distinct from the abnormal distribution seen in the *isa2* mutant (Figs. 3B and C). These results show that the TAP-tagged ISA2 protein is able to restore a functional isoamylase activity and to complement the *isa2* phenotype.

**Figure 4:**
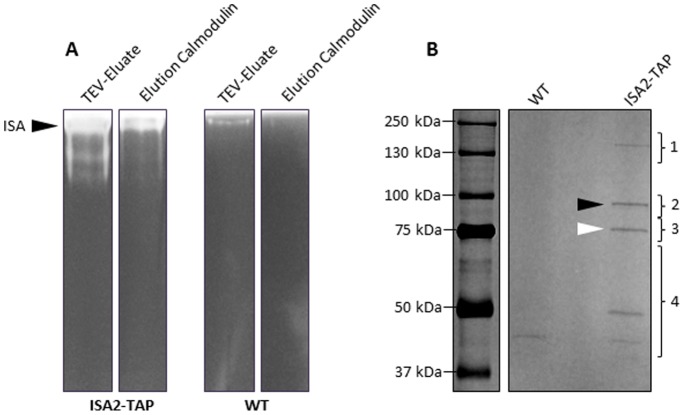
ISA1 co-purifies with ISA2-TAP. A. Amylopectin-containing native gels revealing the isoamylase activity in eluates from the affinity resins during tandem-affinity purification of *ISA2-TAP* and the wild type (WT). The first affinity step is designated ‘TEV-eluate’, and the second ‘Elution Calmodulin’ (see materials and methods).B. Proteins eluting from the calmodulin affinity resin during purification of *ISA2-TAP* and wild-type were concentrated, separated on SDS-PAGE and visualised by silver staining. ISA2-TAP is indicated with the black arrow, ISA1 with the white arrow.

The ISA2-TAP tagged protein was purified from crude extracts of leaves with a two-step affinity purification procedure (see Materials and Methods). The fractions eluted from each purification step were analysed using native PAGE. The purified fraction from *ISA2-TAP* extracts retained isoamylase activity with an electrophoretic mobility comparable to the activity in crude extracts (Fig. 4A). Purifications using extracts of the wild type showed trace amounts of isoamylase activity after the first affinity purification step and none after the second (Fig. 4A). Proteins eluted from the second affinity-purification step were concentrated, separated and visualised by SDS-PAGE (Fig. 4B). From the *ISA2-TAP* extracts, two bands were reproducibly observed corresponding in size to ISA2-TAP itself and to ISA1. In addition, there was a high molecular weight band of around 150 kD found only in the *ISA2-TAP* extracts, and a two smaller bands (40 and 50 kD respectively) that were variable in abundance in different preparations and also observed in extracts of the wild type.

Gel slices containing the protein bands were cut out, subject to in-gel digestion with trypsin, and the peptides analysed by liquid chromatography and tandem mass spectrometry (LC-MS/MS; see Dataset S1). The resulting MS/MS spectra were searched with Mascot version 2.2.04 (Matrix Science) against the Arabidopsis TAIR10 protein database. This confirmed the identities of ISA2-TAP itself and ISA1. The 150 kD band released peptides corresponding to both ISA1 and ISA2, suggesting the formation of a stable dimer. The nature of this dimer remains unclear, as it was not disrupted by the reducing conditions of the SDS-sample buffer and by boiling at 95°C (which should both denature proteins and break any intermolecular disulphide bonds). Analysis of the gel region containing the smaller proteins revealed peptides corresponding to Rubisco, Rubisco activase, and other chaperones. Peptides corresponding to the chloroplastic α-amylase AMY3 were detected in the gel slice containing TAP-ISA2, although AMY3 was not observed in subsequent analyses. We conducted a further two experiments on proteins extracted from batches of independently-grown *ISA2-TAP* plants (see Table S1). Wild-type leaf extracts were treated the same way to serve as controls and identify non-specific contaminants (e.g. very abundant proteins, or proteins interacting with the two affinity resins). In this case, the extracted proteins were initially subject to SDS-PAGE, but only until they reached the resolving gel, whereupon all the proteins in the sample were analysed by in-gel tryptic digestion followed by LC-MS/MS. These experiments confirmed the enrichment of ISA1 with ISA2-TAP, which were the most abundant proteins with comparable numbers of peptides derived from each. No other protein was consistently detected as enriched in the ISA2-TAP samples. These data suggest that the ISA1–ISA2 enzyme does not form stable associations with other proteins.

**Figure 5:**
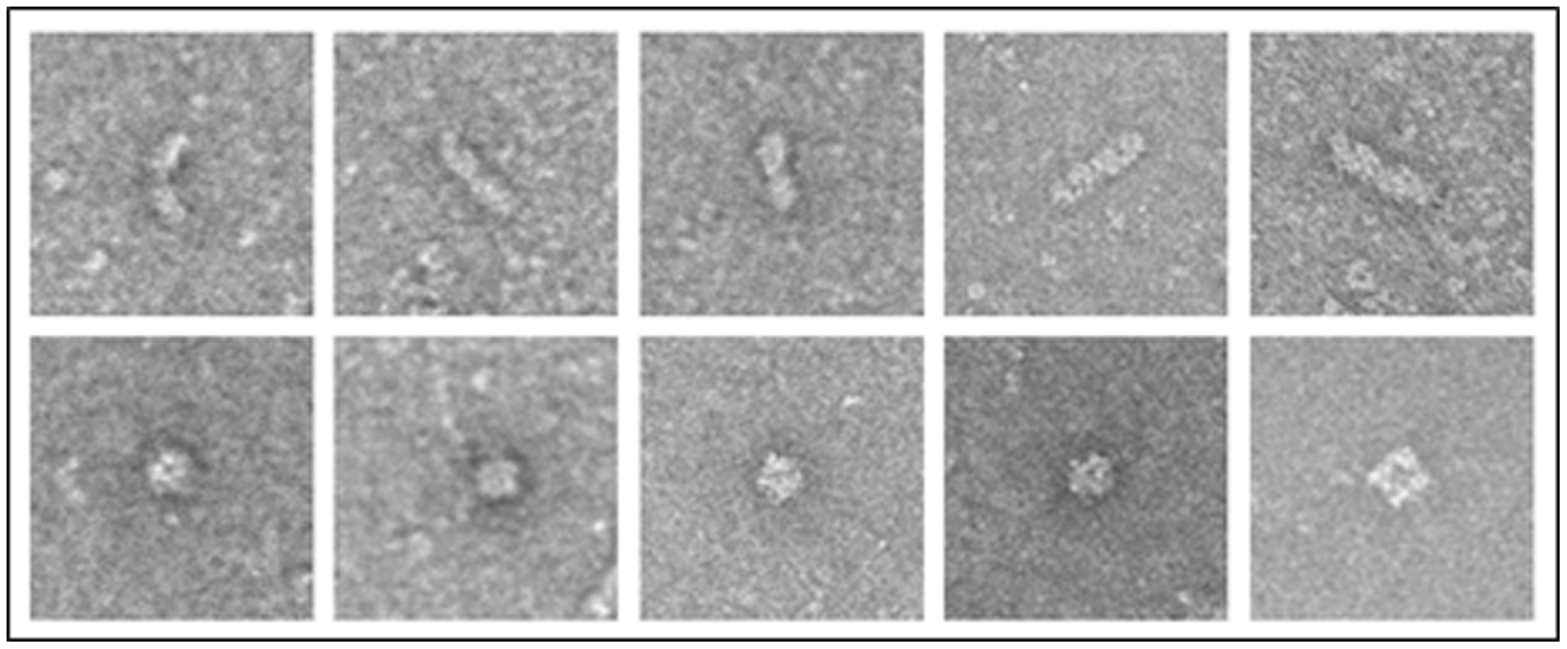
Molecular structures the affinity purified ISA2–TAP complex. Purified ISA2-TAP protein was spotted onto carbon grids, negatively stained with uranyl acetate and analysed by transmission electron microscopy. The most abundant particles were rod-like, or in some cases dumbbell-like (upper images). In additional globular particles were observed, consistent with the presence of some residual RUBISCO contamination (lower images). In each case the field of view is 80 nm × 80 nm.

As the TAP-ISA complex was nearly pure, albeit low in yield, we analysed its molecular structure with transmission electron microscopy. Rod-shaped particles approximately 30 nm length and globular particles approximately 10 nm in diameter were observed in the micrographs (Fig. 5). The globular particles appeared similar to the known protein structure of Rubisco [23]. The rod-shaped particles probably represent the ISA1–ISA2 complex. Some of the rod-shaped particles had a dumbbell-like appearance, suggesting that the native enzyme may be a dimer of dimers.

### The ISA1–ISA2 Enzyme Shows Distinct Branch Specificity during α-polyglucans Hydrolysis

**Figure 6:**
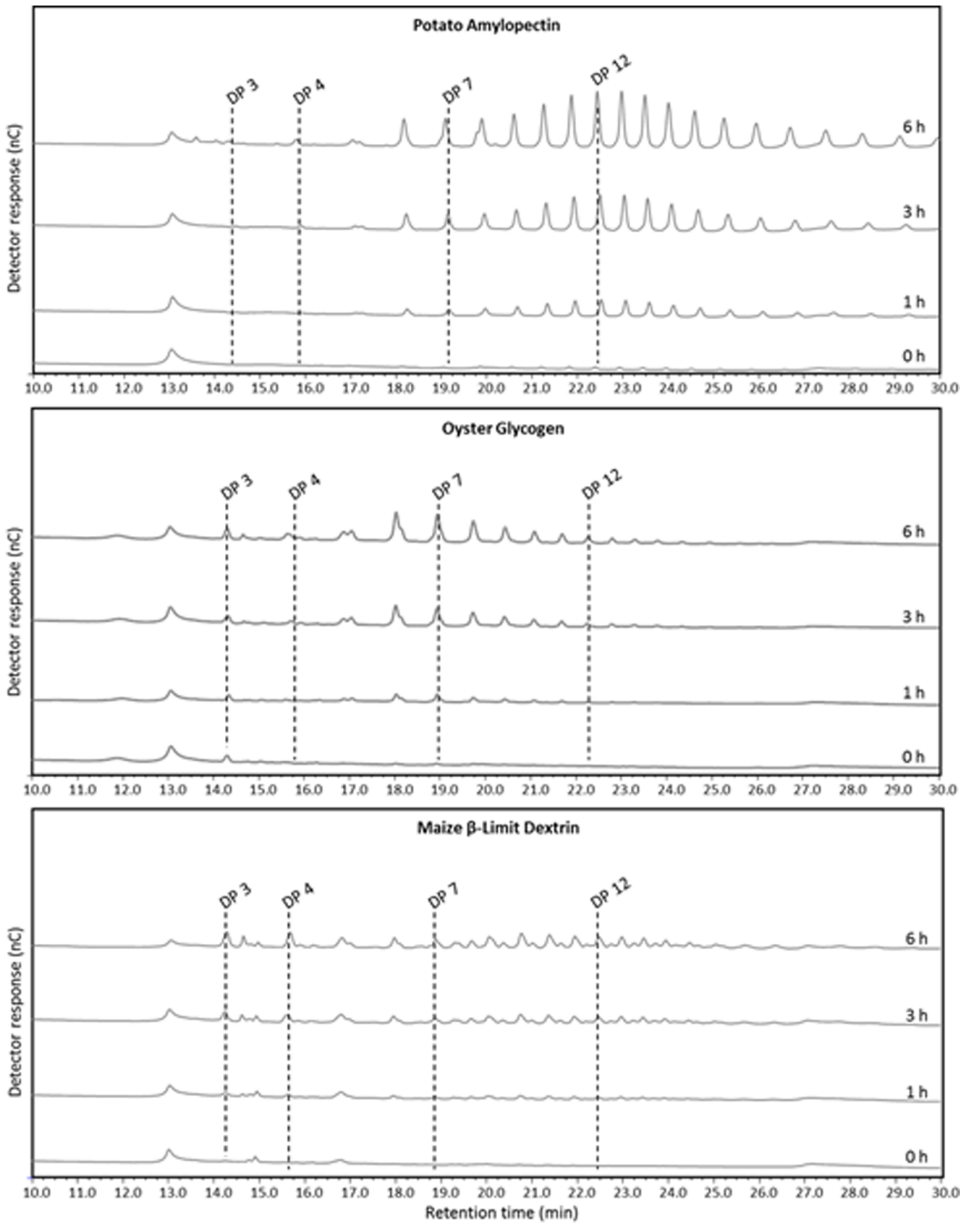
Activity of the ISA1-ISA2 isoamylase against different branched glucan substrates. Potato amylopectin, oyster glycogen or maize β-limit dextrin (200 µg) was digested with partially purified ISA1–ISA2-TAP at pH 7.2 and 30°C. At different time points, aliquots were withdrawn and adjusted to 0.1 M NaOH to stop the reaction. Liberated glucan chains were separated and detected by HPAEC-PAD. Note that digestion of the β-limit dextrin gives rise to additional peaks that represent branched malto-oligosaccharides.

To explore the chain-length specificity of tandem affinity purified ISA1–ISA2, the enzyme from the first affinity step was incubated with potato amylopectin, oyster glycogen and maize β-limit dextrin and the liberated chains were analysed by HPAEC-PAD. Amylopectin and glycogen were debranched by the purified enzyme into linear chains with typical chain-length distributions (Fig. 6, A and B). Interestingly, we earlier provided indirect evidence with crude extracts of *amy3*/*isa3*/*lda* mutants that, when faced with a β-limit dextrin, ISA1–ISA2 releases predominantly branched oligosaccharides [24]. This was confirmed by analysis of the products of hydrolysis of β-limit dextrin by the tandem affinity purified ISA1–ISA2. Major and minor peaks were observed in the HPAEC-PAD chromatograms, corresponding to a mixture of linear oligosaccharides and branched oligosaccharides, with the latter containing up to 16 glucose residues (Fig. 6C). The experiment was also performed with elution sample from the second affinity purification step with comparable results (not shown).

### Expression of Recombinant ISA1 and ISA2 in *E. coli*

**Figure 7:**
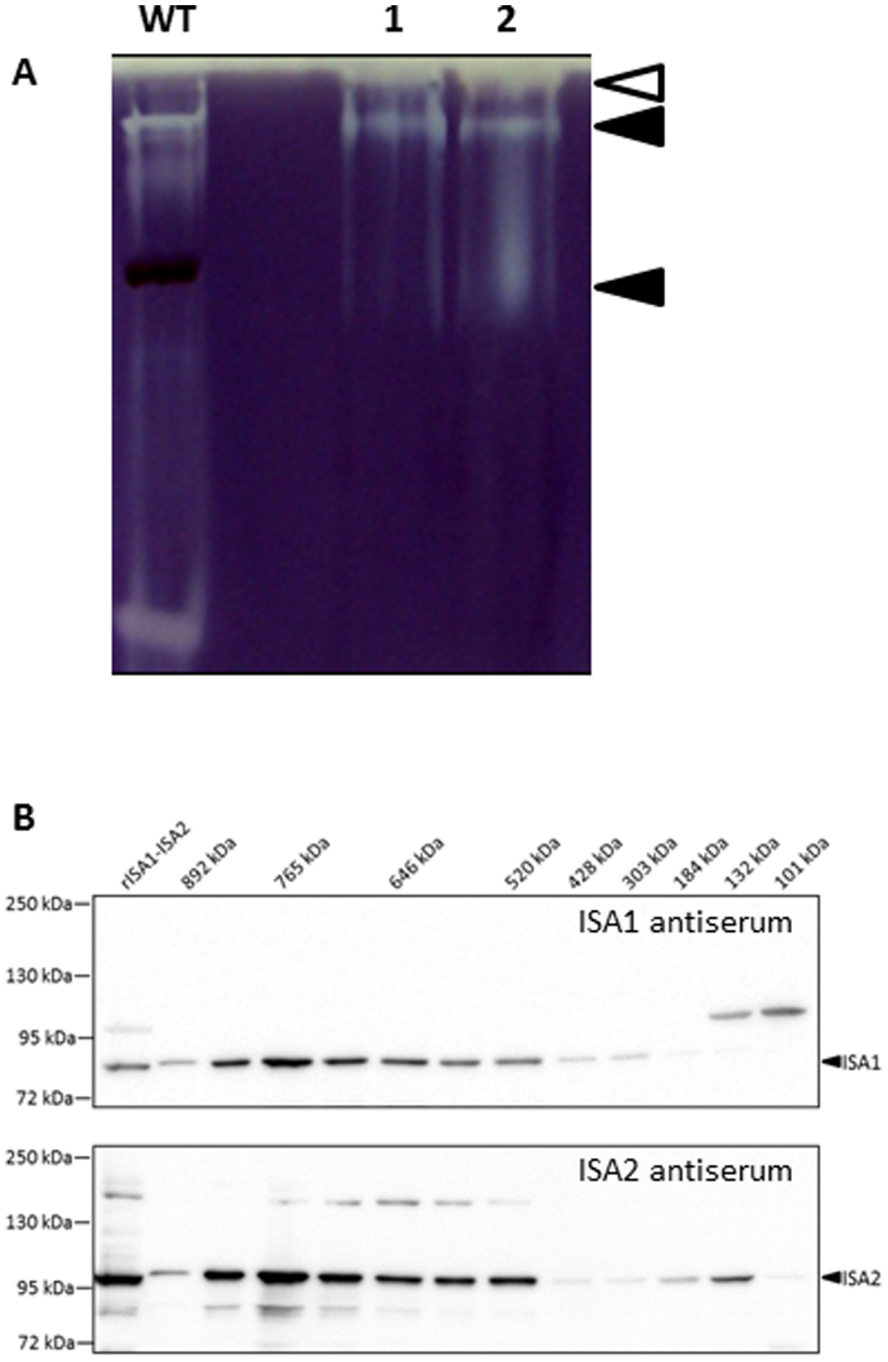
Recombinant expression of the ISA1–ISA2 isoamylase. A. Soluble protein lysates from *E. coli* cells co-expressing different expression vector combinations of ISA1 and ISA2 were analysed by native PAGE in amylopectin-containing gels. The vector combinations are: 1, p0GWA-ISA1+ pET29a-ISA2 and 2, pET29a-ISA1+ p0GWA-ISA2. An extract of wild-type Arabidopsis leaves is shown for comparison (WT). The black arrow marks ISA1-ISA2 activity and the white arrow marks a background activity from *E. coli* lysate. B. Gel filtration chromatography of extracts of *E. coli* cells co-expressing ISA1 and ISA2. Fractions from a Sephacryl HiLoad 200 prep grade column were collected, concentrated and separated by SDS-PAGE. ISA1 and ISA2 were detected by immunoblot analysis probed with specific antibodies. The molecular masses based on the elution positions of known standards are indicated. A crude extract of *E. coli* expressing the recombinant isoamylase is shown on the left for comparison (rISA1–ISA2).

Recombinant ISA1 and ISA2 from potato were previously expressed in *E. coli*, as S-tagged proteins and StISA1 shown to have isoamylase activity [13]. Encouraged by these reports, we expressed Arabidopsis ISA1 and ISA2 proteins individually, purified them, and mixed them together to try and reconstitute the complex. Several expression vectors were tried, of which p0GWA yielded ISA proteins in the soluble fraction (not shown). However, using native PAGE, no activity was detectable for the individual isoforms, nor when mixing the two partially purified recombinant proteins (not shown). Therefore, we tested if co-expression from separate vectors in the same *E. coli* cells would produce an active complex. For this, the vector pair p0GWA (containing the ampicillin resistance gene) and pET29A (containing the kanamycin resistance gene), each of which contained either ISA1 or ISA2 was co-transformed into *E. coli* BL21 Codon-Plus. Both combinations (p0GWA-ISA1:pET29a-ISA2 and p0GWA-ISA2:pET29a-ISA1) resulted in DBE-activity on native PAGE gels, with the recombinant proteins migrating in a similar way to the native enzyme in Arabidopsis leaf extracts (Fig. 7A). Gel filtration chromatography revealed that the recombinant ISA1 and ISA2 eluted together in fractions corresponding to molecular weights ranging from 800-520 kDa but with a peak for both proteins at 750 kDa (Fig. 7B). This is significantly larger that the native ISA1–ISA2 complex found in Arabidopsis of 430 kDa (Fig. 2C). The reason for this apparent difference, despite the comparable mobility in the native PAGE is unclear.

Important amino acid residues were identified by aligning ISA1 and ISA2 sequences from various plant species with the *Pseudomonas amyloderamosa* isoamylase (PaISO), for which a crystal structure is available (PDB ID: 1BF2) [25]. Eight residues reported to be absolutely conserved in all active members of the α-amylase family lie within the active-site: Asp-292, Val-294, His-297, Arg-373, Asp-375, Glu-435, His-509 and Asp-510 (numbering according to PaISO, minus its 26 amino acid signal peptide). The three carboxylic acid residues Asp-375, Glu-435 and Asp-510 are essential for catalytic activity [26]. ISA1 has been proposed to be the catalytic subunit of the ISA complex [13] and all eight residues are conserved in the Arabidopsis ISA1 sequence (Fig. S1). In the Arabidopsis ISA2 sequence, only Val-294 and His-297 are conserved. The remaining six residues differ from ISA1 as follows; Asp-292 → Glu, Arg-373 → Cys, Asp-375 → Ile, Glu-435 → Asp, His-509 → Asn, and Asp-510 → Ser. Interestingly, the ISA2 sequences from maize and rice share the exact same substitutions. Based on comparable substitutions in the potato ISA2 protein, Hussain et al. (2003) proposed that ISA2 is a hydrolytically inactive. To test the role of ISA1 as the catalytically active subunit, site-directed mutagenesis was performed to substitute the predicted catalytic nucleophile Asp-367 in ISA1 with an alanine (D367A, according to the ISA1 sequence after removal of its predicted transit peptide, equivalent to Asp-375 in the Pseudomonas enzyme). The D367A mutant protein was co-expressed with non-mutated ISA2. SDS-PAGE confirmed that the D367A protein was present in comparable amounts as the non-mutant version (hereafter called *active* ISA). As expected, the mutation abolished catalytic activity on native gels (hereafter called *inactive* ISA).

### Impact of the Recombinant ISA1–ISA2 Complex on Glycogen Structure

**Figure 8:**
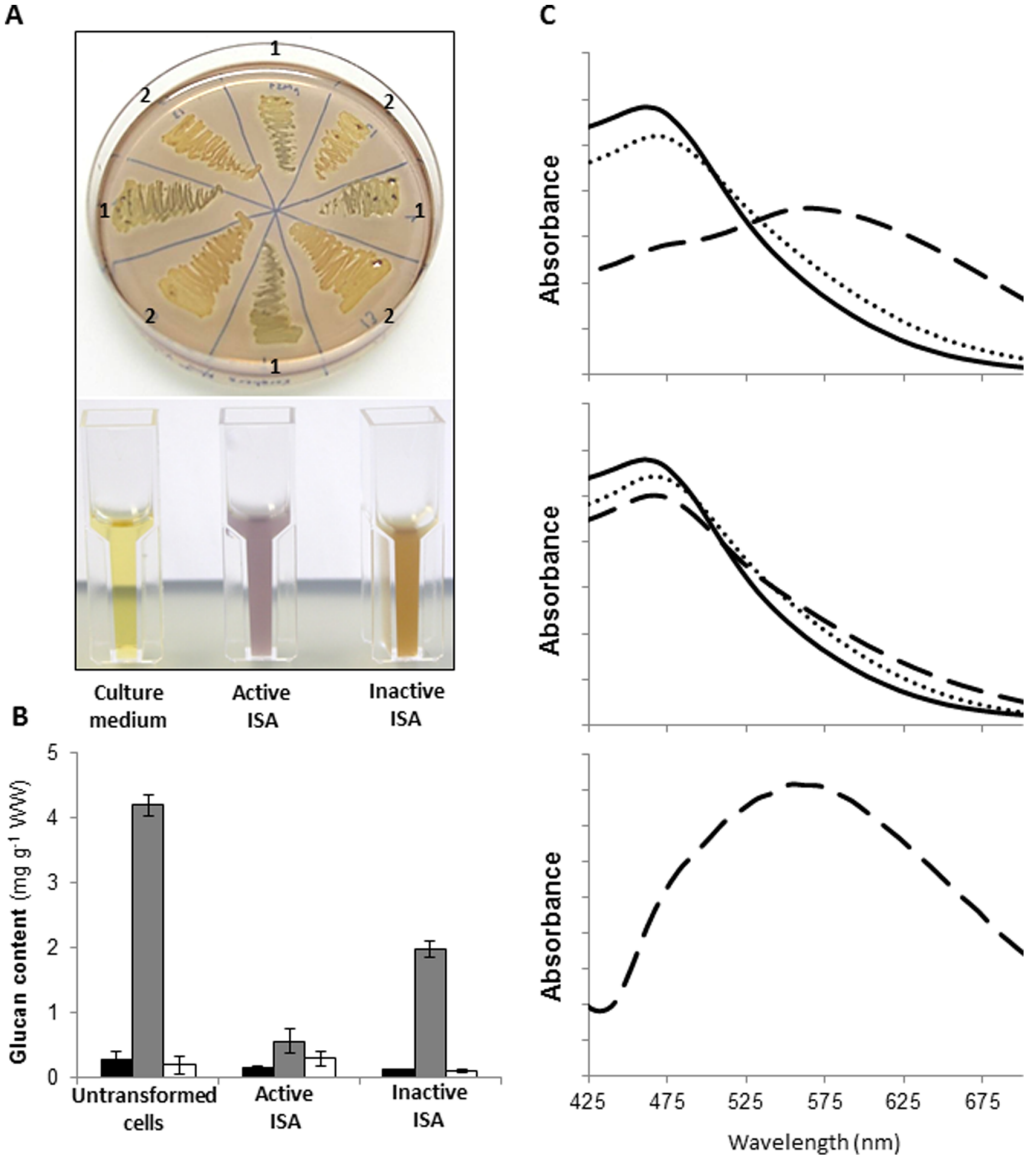
Expression of the recombinant ISA1–ISA2 isoamylase alters glycogen biosynthesis in *E. coli*. A. Glucan content per gram wet weight (WW) of untransformed *E. coli* cells and transformed cells expressing either the active or inactive isoamylase complex. Black bars, insoluble glucan; Grey bars, methanol-precipitable soluble glucan; white bars, non-precipitable soluble glucan. Values are the means ± the standard error from measurements from duplicate experiments. B. *E. coli* cells expressing either the active (1) or inactive (2) isoamylase complex were grown on solid medium (top) and stained with iodine. Note the dark staining of cells expressing the active enzyme. Similar staining is observed with cells grown in liquid medium (bottom). C. Absorption spectra of the glucan-iodine complexes from untransformed *E. coli* cells (solid line) and transformed cells expressing either the active (dashed line) or inactive (dotted line) isoamylase complex. Top panel: methanol-precipitated glucans from the soluble fraction. Middle panel: glucans retained after ultrafiltration using a 50 kDa molecular weight cut-off filter. Lower panel: filtrate from the 50 kDa molecular weight cut-off filter.

When grown to stationary phase in minimal media in the presence of an excess of carbohydrate, *E. coli* produces glycogen [27]. Therefore, we tested whether the expression of the active ISA altered the glycogen structure in a way that would help to explain its role starch biosynthesis. First, cells were grown on solid Kornberg medium and stained with iodine: those expressing the active ISA were dark blue-green whereas those expressing the inactive ISA stained red-orange (Fig. 8A), comparable to the staining of wild-type glycogen (not shown). These initial data suggested that the presence of the active ISA does indeed alter glycogen structure, resulting in a glucan that stains more like starch. When the same clones were grown in liquid Kornberg medium the staining pattern was even more distinct (Fig. 8A).

We used several techniques to further investigate the structure of these glucans. First, cells were fixed, embedded in resin, and analysed by transmission electron to determine whether any starch-like insoluble glucan could be observed. However, the cells expressing either the active or the inactive ISA were indistinguishable and did not contain any starch-like structures (Fig. S2). Next, the quantity of soluble and insoluble glucans was determined in cell extracts. Essentially all of the glucans were found in the soluble phase in cells expressing either the active or the inactive ISA, and in non-transformed cells. The highest glucan levels were observed in the non-transformed cells, in which no protein biosynthesis was induced. Interestingly, despite the intense staining of the cells expressing the active ISA, there was a major reduction in glucan content compared with cells expressing the inactive ISA (Fig. 8B). This is possible as the iodine-binding capacity and staining intensity of glucans is highly dependent on the secondary structures that they adopt.

**Figure 9:**
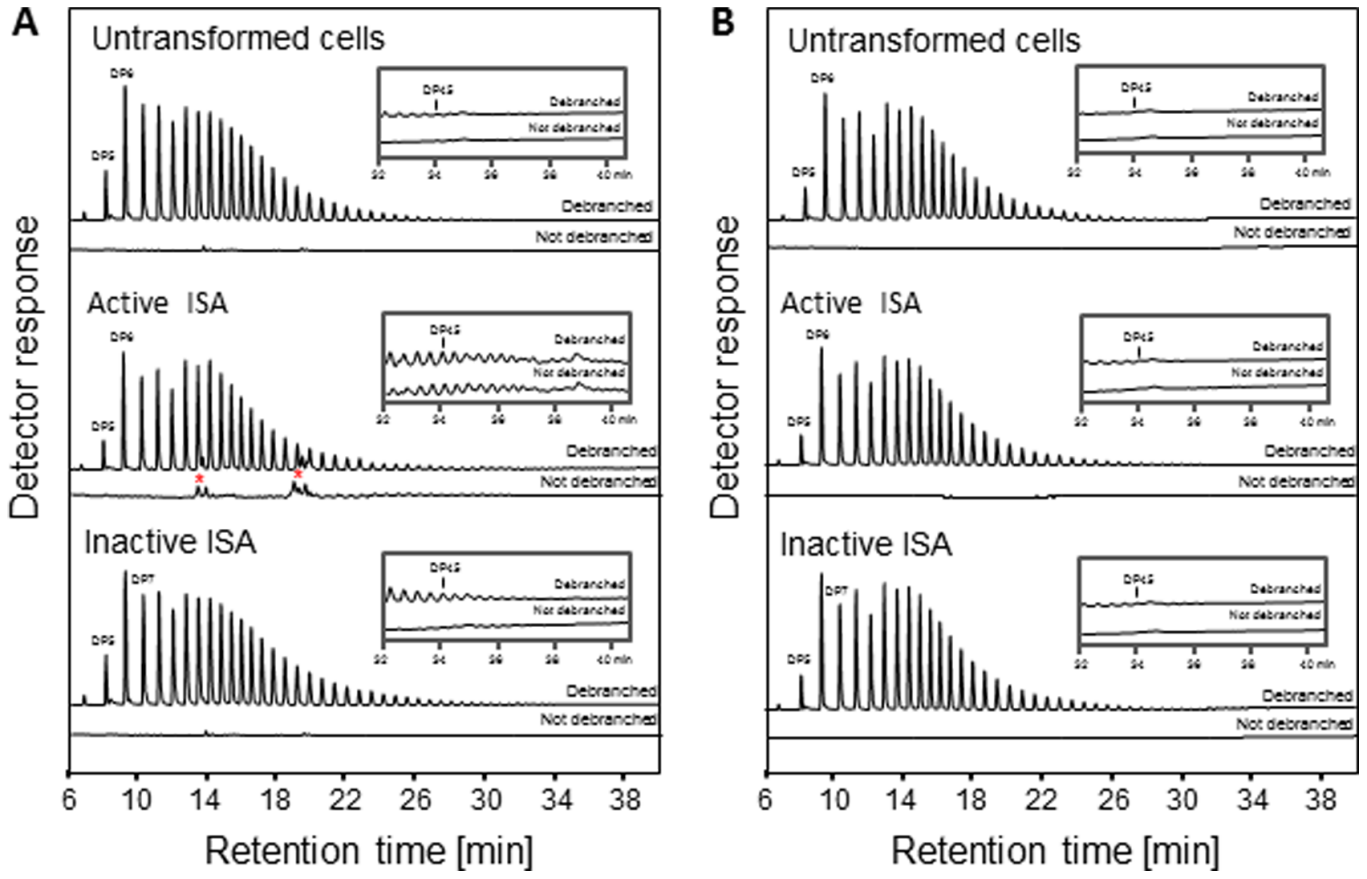
Chain-length analysis of glucans synthesised in *E. coli* in the presence or absence of the ISA1–ISA2 isoamylase. A. Methanol-precipitated glucans from the soluble fraction from of untransformed *E. coli* cells and transformed cells expressing either the active or inactive isoamylase complex were analysed by HPAEC-PAD, with or without a preceding debranching step. Note the presence of glucans between d.p. 20 and 50 in the non-debranched fraction of cells expressing the active isoamylase but not of the other lines (inset). Peaks marked with a red asterisk derive from *E. coli* itself and are not α-1,4−/α-1,6-linked glucans, judged by their resistance to digestion by amyloglucosidase (not shown). B. Methanol-precipitated glucans as in [A] were subject to ultrafiltration using a 50 kDa molecular weight cut-off filter. The glucans retained by the filter were analysed as in [A]. Note that the glucans between d.p. 20 and 50 that were present in the non-debranched fraction of cells expressing the active isoamylase are now absent, having passed through the filter.

Most of the glucan in the soluble extracts of all three lines could be precipitated by the addition of methanol. We redissolved the precipitated glucans and obtained absorption spectra for each when complexed with iodine. The glucans from both the non-transformed cells and from the line expressing the inactive ISA had a peak absorption (λ_max_) of 460 nm, typical of glycogen. In contrast, the glucan from the line expressing the active ISA had a λ_max_ between 550 and 600 nm (Fig. 8C). We analysed the chain length distribution (CLDs) of these precipitated glucans by HPAEC-PAD after debranching them with commercial debranching enzymes. CLDs typical of glycogen were obtained for all three lines (Fig. 9A). Surprisingly, the distributions differed only slightly, with the line expressing the active ISA slightly enriched in longer chains with a degree of polymerization (d.p.) of between 20 and 50.

We repeated the analysis omitting the debranching enzyme pre-treatment. No glucans were detected in the non-treated glucan samples of untransformed *E. coli*, or the line expressing the inactive ISA (intact glycogen is too large to be detected by HPAEC-PAD). In contrast, the line expressing the active ISA contained a population of oligosaccharides between d.p. 20 and 50 (Fig. 9B). We speculated that these oligosaccharides may be separated from glycogen by size. Therefore, we subjected them to ultrafiltration using a 50 kD cut-off filter – a pore size small enough to retain glycogen, but large enough to allow malto-oligosaccharides to pass through. We stained both the retentate and the filtrate with iodine. Red-staining glycogen was retained in all three samples (λ_max_ = 460 nm: Fig. 8D). The filtrate of the sample from untransformed *E. coli* and the line expressing the inactive ISA did not stain, whereas the filtrate of the line expressing the active ISA stained blue (λ_max_ = 580 nm: Fig. 8E). We re-analysed the CLD of the filter-retained glycogen from the line expressing the active ISA. It was similar to that of the untransformed *E. coli* and the line expressing the inactive ISA, and was not enriched in chains between d.p. 20 and 50. This suggests that the ISA1–ISA2 enzyme releases oligosaccharides from glycogen. These oligosaccharides may themselves be further elongated by glycogen synthase to form a population of long, linear or lightly branched chains.

We also examined the chain length distribution after pre-treating the glucans with β-amylase to degrade external, unbranched glucan chains (βCLDs). Subsequent debranching of the resultant β-limit dextrin gives a measure of the branch point distribution of the glucan ([28]. However, no differences were observed between the βCLDs of the glucans from the three strains (not shown).

Together, these data show that the presence of the ISA1–ISA2 enzyme does not in fact have a major impact on glycogen chain length distribution, but rather causes its partial degradation, resulting in the accumulation of low molecular weight malto-oligosaccharides which stain intensely with iodine, presumable after adopting a single helical conformation comparable to that taken by amylose.

## Discussion

This work provides valuable insight into the nature of the isoamylase enzyme involved in starch biosynthesis in Arabidopsis. Our data are consistent with the idea that the isoamylase is present *in vivo* only as a heteromultimer. This is based on several observations. Firstly, comparable amounts of ISA1 and TAP-ISA2 proteins were recovered after the affinity purification experiments, suggesting a stoichiometry of 1∶1, as reported for the potato enzyme [15]. Secondly, although the native size (approximately 430 kDa) of the wild-type enzyme is slightly larger than that predicted for a tetramer of two ISA1 and two ISA2 subunits, the electron microscopy images of negatively-stained, affinity-purified enzyme suggests that it adopts an elongated ‘dumbbell’-like structure. This might migrate more slowly in a gel-filtration medium than a globular protein of comparable molecular weight. We speculate that the complex is made up of a dimer of ISA1–ISA2 heterodimers. If ISA1–ISA2 heterodimers are themselves active, it might explain the appearance of the second, faster migrating band when both proteins are together expressed as recombinant proteins in *E. coli* (Fig. 7A). We cannot exclude the possibility that the enzyme is a heterodimer of ISA1 and ISA2 homodimers, although we do not favour this idea because we were unable to detect any activity when expressing ISA1 alone. However, ISA1 from other species has been successfully expressed as a recombinant fusion protein [15], so further experiments will be needed to resolve this question. The fact that the TAP-tagged ISA complex can be enriched to near purity, albeit in small amounts, will allow further electron-microscopy-based approaches to an improved structure of the complex.

Our TAP-tagging approach also allowed us to search for potential interaction partners amongst the other starch metabolic enzymes. The near-complete complementation of the *isa2*-1 mutant phenotype showed that the tagged ISA2 protein was functioning normally in starch biosynthesis. Despite this, we found no evidence for direct interaction between the isoamylase and any starch biosynthetic or degradative enzymes. This is consistent with other studies. Complexes between starch synthases and starch branching enzymes have been reported in the developing endosperms of maize and wheat, but in neither case has interaction with isoamylases been reported [29], [30].

Our results suggest strongly that ISA1 and ISA2 are mutually dependent on each other for stability *in vivo*, as well as for activity. Our results confirmed the previous observation that ISA1 protein is decreased in absence of ISA2, and no activity is detectable [10]. Furthermore, we were unable to detect the ISA2 protein in the *isa1* mutant. The fact that each gene was still expressed normally when in plants mutated for the other gene, suggests that the translated proteins are degraded when they cannot form a complex. This situation differs from that in the endosperms of several Poaceae species. In maize and rice, ISA1 is able to form both homomultimers and multiple heteromultimers with ISA2 [16], [17]. The homomultimers are stable in the absence of ISA2 and are believed to be most active in starch biosynthesis [16], [17]. However, the heteromultimer was reported to be more stable at higher temperatures [17], and it is interesting to note that only heteromultimeric forms were observed in rice leaves. The basis for these differences in subunit composition and function in starch biosynthesis between isoamylases from different species still needs to be resolved.

The expression of the ISA1–ISA2 enzyme has a marked impact on the formation of glycogen in *E. coli.* Initial iodine staining suggested that the enzyme’s presence resulted in a darker-staining and perhaps more amylopectin-like polymer, rather than glycogen. This was also seen for the expression of the potato ISA1 protein alone [13]. However, further analysis revealed that this dark staining is due to the presence of long malto-oligosaccharides, which are presumably able to adopt a single helical structure that tightly binds iodine, as would amylose. This explains the intense staining despite the overall reduction in glucan amount. Chains of d.p. 20 to d.p. 50 are relatively rare in glycogen. Thus, the ISA1–ISA2 isoamylase probably removes shorter chains from glycogen, resulting in a pool of linear or lightly branched oligosaccharides that are elongated by glycogen syntheses. This hypothesis is consistent with the fact that glycogen was also present in cells expressing ISA1–ISA2 at lower amounts than in the other lines, but that its structure is similar to that made by untransformed *E. coli* cells or cells carrying the catalytically dead enzyme. It is important to note that these experiments were carried out in the presence of the endogenous *glgX* gene, which encodes an isoamylase. This enzyme is primarily involved in degradation, as mutants lacking the GlgX protein accumulate more glycogen than wild-type cells. However, it also affects glycogen structure as the glycogen from mutant cells is enriched in very short chains of d.p. 3 and d.p. 4, for which this enzyme is specific [31]. However, overexpression of GlgX [31] was not reported to cause a similar phenotype to that resulting from the expression of the ISA1–ISA2 enzyme reported here. GlgX is probably more similar in its function to the ISA3 isoform of higher plants, which also preferentially removes short chains [12], [18].

Our data raise an important question about the function of the ISA1–ISA2 isoamylase. ISA1–deficient plants have a marked change in glucan structure, implicating the enzyme in biosynthesis. However, genetic studies show that the ISA1–ISA2 enzyme is seemingly ineffective in contributing to starch breakdown: Streb et al. [24] showed that mutants lacking the three enzymes isoamylase 3, limit dextrinase and α-amylase 3 are unable to degrade any starch, despite the presence of ISA1–ISA2. Yet the recombinant ISA1–ISA2 enzyme is clearly active and able to degrade glycogen, solubilised amylopectin and, to some extent, β-limit dextrin. In *E. coli*, the enzyme seems primarily to degrade glycogen rather than modify its structure. This suggests that for ISA1–ISA2 to fulfil its role in starch biosynthesis, it needs to be active in the right context. For example, the branched glucan on which it acts in a plant cell is the product of multiple biosynthetic enzymes (starch synthase and branching enzyme isoforms), with which it has co-evolved. Thus, ISA1–ISA2 may be able to debranch this ‘pre-amylopectin’ effectively to promote its crystallization into a starch granule. However, when faced with glycogen in *E. coli*, its debranching action may not result in a crystallization-competent structure – rather it just serves to decrease glycogen accumulation by counteracting the glycogen biosynthetic enzymes. A comparable concept has been suggested to explain the importance of the ISA1 homomultimers in rice endosperm, relative to the ISA1–ISA2 heteromultimers. In the endosperm, ISA2 is present, but not required for normal starch biosynthesis, which can be mediated by ISA1 homomultimers alone. Over-expression of ISA2 titrates out the ISA1, but the resulting ISA1–ISA2 heteromultimers cannot mediate normal starch synthesis. One proposed explanation is that the homomultimers and the heteromultimers have differing specificities and only the specificity of the homomultimers matches the synthase/branching enzyme combinations expressed in the endosperm [17].

Although we feel this is the most plausible explanation, others exist: the enzyme in plants may be subject to post-translational modifications, not replicated in *E. coli*, that influence its activity. However, no such modifications have been reported to date. Another possibility is that the stoichiometry of the respective enzymes is important. The right balance of elongating, branching and debranching activities may be important as well as - or more than - their specificities. Thus, it is possible that too much ISA1–ISA2 protein is expressed in our *E. coli* experiments. We feel this is unlikely to be the full explanation, since one would still expect a change in glycogen structure in addition to the decrease in glycogen amount we observed. However, further work with combinations of expressed enzymes, either *in vitro* or in a heterologous system, should allow both stoichiometry and specificity to be evaluated as determinants of glucan structure.

## Materials and Methods

### Plant Material and Growth Conditions

*Arabidopsis thaliana* (Col-0 accession) plants used for metabolite measurements and tandem affinity purification were grown in a Percival AR95 growth chamber (Percival Scientific Inc., Perry USA), with a 12-h light/12-h dark diurnal cycle, a uniform light intensity of 150 µmol quanta m^−2^ s^−1^, 70% relative humidity, and a constant temperature of 20°C. Seeds were sown out on fine-grade soil and stratified at 4°C in the dark for two days. Ten-day old seedlings were transferred to individual 200-mL pots filled with nutrient-rich, medium-grade, peat-based soil.

### Protein Native Molecular Weight Determination by Gel Filtration Chromatography

An ÄKTA Explorer equipped with a Sephacryl HiLoad 200 prep grade column (GE Healthcare, Glattbrugg, Switzerland) was used for molecular weight determination. The column was calibrated with a Gel Filtration HMW Calibration Kit (GE Healthcare) according to the manufacturer’s instructions. Soluble proteins were extracted from Arabidopsis leaves or *E. coli* cells in an ice-cold medium containing 100 mM Tris-HCl, pH 7.5, 300 mM KCl, 5 mM DTT. All subsequent steps were performed at 4°C. Five hundred microliter protein sample was injected with a sample loop onto the column, and proteins were eluted in the same buffer at a flow of 0.5 mL min^−1^. Fractions (2 ml) were collected, divided into 200-µL aliquots, frozen in liquid N_2_ and stored at −80°C. Prior to SDS-PAGE and immunoblot analysis, proteins were concentrated by methanol precipitation at −20°C.

### Native PAGE

Soluble proteins from Arabidopsis leaves were extracted in 50 mM 2-amino-2-hydroxymethyl-propane-1,3-diol (Tris)-HCl, pH 7.25, 10% (v/v) glycerol, 1 mM ethylenedinitrilo-tetraacetic acid (EDTA) and 5 mM dithiothreitol (DTT). Proteins produced in *E. coli* were extracted in 50 mM Tris-HCl pH 7.25, 150 mM NaCl, 5 mM DTT. Aliquots of the supernatants were mixed with loading buffer (50% [v/v] glycerol, 0.05% [w/v] Bromophenol Blue) in a ratio of 5∶1 prior to loading onto 6% (w/v) polyacrylamide gels, containing 0.1–0.2% (w/v) potato amylopectin (Sigma-Aldrich) or maize β-limit dextrin (Sigma-Aldrich), and separated at 100 V for 3 h at 4°C. After electrophoresis, gels were incubated in 100 mM Tris-HCl, pH 7.2, 1 mM MgCl_2_ and 1 mM CaCl_2_ for 2 h at 37°C. Amylolytic activities were visualised by staining the gels in Lugol solution (Sigma-Aldrich, Buchs, Switzerland) followed by destaining in cold water.

### General and Molecular Methods

Plant DNA was extracted using the GenElute Plant genomic DNA miniprep kit according to the manufacturer’s instructions (Sigma-Aldrich). Bacterial DNA was extracted and purified with NucleoSpin® Plasmid QuickPure (Macherey-Nagel, Oensingen, Switzerland). Purified DNA was quantified using a NanoDrop ND-1000 Spectrophotometer (NanoDrop Technologies, Wilmington, USA).

### Quantitative PCR (qPCR)

Leaves from 3 individual 20-day old plants were harvested at the end of night, pooled and immediately frozen in liquid N_2_. RNA was extracted using Spectrum™ Plant Total RNA kit (Sigma-Aldrich) according to the manufacturer’s instructions. Following on-column DNase treatment (Roche, Rotkreuz, Switzerland), RNA was reverse-transcribed to cDNA using SuperScript® III Reverse Transcriptase (Invitrogen, Basel, Switzerland) according to the manufacturer’s instructions. For qPCR, Fast SYBR Green Master Mix (Applied Biosystems, Rotkreutz, Switzerland) was used on a 7500 Fast Real-Time PCR system (Applied Biosystems). PP2A (At1g13320) was use as a constitutively expressed control. Primer sequences for qPCR were as follows: PP2A, forwards 5′-TAA CGT GGC CAA AAT GAT GC-3′, reverse 5′-GTT CTC CAC AAC CGC TTG GT-3′; ISA1, forwards, 5′-GATCAGATACGCATCAGCA-3′, reverse 5′-TCCATGATTACCTCAATTCCTC-3′. ISA2, forwards 5′-GCAAATGACCAAACAACTCC-3′, reverse 5′-GACGCAGTTTCCTCTTCC-3′.

### Creation of the ISA2-TAP Tag, Plant Transformation and Complementation Analysis

The *ISA2* coding sequence of without its stop codon was fused to a sequence encoding a 20 kDa C-terminal tandem affinity purification (TAP) tag, comprising a calmodulin-binding peptide (CBP), a tobacco etch virus (TEV) protease cleavage site and two IgG binding domains of Protein A from *Staphylococcus aureus*, based on the construct from Rigaut et al. [22]. The *ISA2-TAP* sequence was cloned into the multiple cloning site of the binary vector pGreenII 0179 [32] behind the cauliflower mosaic virus 35 S promoter. The construct was transformed into Arabidopsis *isa2*-1 mutants using Agrobacterium [33]. Transformants were selected on Murashige and Skoog (MS) plates containing 1% agar and 25 mg/l hygromycin. For complementation analysis, several independent transformed lines were germinated on selective MS-plates alongside the respective wild-type and mutant controls (on non-selective plates). After transplantation onto soil, plants were harvested and extracted with perchloric acid as described previously [10]. Starch in the insoluble fraction and phytoglycogen in the soluble fraction were determined as described previously [34]. Structural analysis of starch and phytoglycogen by HPAEC-PAD was performed as described previously [10].

### Tandem Affinity Purification Procedure

Tandem affinity purification was performed as previously described [21], [22] with minor modifications. Wild-type and ISA2-TAP leaf material harvested at the end of the day was pulverised in liquid N_2_ with a mortar and pestle, then homogenised in glass homogenisers with ice-cold extraction buffer (50 mM Tris-HCl, pH 7.25; 150 mM NaCl, 5% [v/v] glycerol, 5 mM EDTA, 5 mM DTT and 1×Protease Inhibitor [Roche]). Homogenates were clarified by centrifugation (20 000 *g*, 4°C, 15 min) and the supernatant filtered through 0.45 µm filters. The tagged protein and associated components were recovered by incubation of the extract with 500 µL IgG-Sepharose 6 Fast Flow beads (GE Healthcare) per 5 g starting material for 2 h at 4°C. After washing, 100 units Tobacco Etch Virus protease (AcTEV™ Protease, Invitrogen) in TEV buffer (50 mM Tris-HCl, pH 8.0, 0.5 mM EDTA, 1 mM DTT) was added and the suspension was agitated gently for 1 h at 25°C to cleave off the bound proteins. The supernatant was incubated with Calmodulin Sepharose 4B (GE Healthcare) in the presence of 2 mM CaCl_2_ for 16 h at 4°C with gentle agitation to remove the TEV protease and trace contaminants remaining after the first affinity selection. After washing, the bound proteins were released by chelating the calcium with EGTA. As a negative control, extracts of the wild-type were processed through all stages of the tandem affinity purification procedure.

### Mass-spectrometric Analyses of Tandem-affinity-purified Protein Samples

Protein samples obtained from TAP-tagging experiments were subjected to SDS-PAGE on 10% (w/v) gels. Gels were fixed for 1 h at 25°C in 12% (v/v) trichloroacetic acid and stained for 16 h with 0.1% (w/v) Coomassie Blue G-250 in 20% (v/v) methanol, 10 mM ammonium sulphate, 2 mM orthophosphoric acid, followed by destaining in distilled water. Bands of interest were cut out and the gel slice was diced into small pieces. In-gel digestion was performed as described previously [35] with modifications. Details of the peptide analytical procedure are given in Dataset S1.

### Electron Microscopy

Protein samples from the second TAP-elution were concentrated in 500-µL Vivaspin columns (Sartorius, Tagelswangen, Switzerland) and 4 µL were applied to 400 MESH carbon grids. The grids were washed several times on a water-droplet, then negatively stained with uranyl acetate and analysed in a Philips CM 12 transmission electron microscope coupled to a CCD-camera.

*E. coli* cells were fixed directly in the culture medium with 2% (v/v) glutaraldehyde for 1 h at 20°C. Samples were washed three times in ice-cold 0.1 M sodium cacodylate buffer, pH 7.4. The pellets were post-fixed in 1% (w/v) aqueous osmium tetroxide, 0.1 M sodium cacodylate buffer, pH 7.4 for 2 h at 4°C, then washed three times in sodium cacodylate buffer and once in water. After wash, the cells were dehydrated in an ethanol series and infiltrated and embedded in epoxy resin (Spurr’s, Agar Scientific, Stansted, UK) using gelatin capsules. The capsules were centrifuged (1000 g, 5 min) to collect the cells at the bottom of the capsules. Ultra-thin sections were cut with a diamond knife and stained sequentially with uranyl acetate and Reynold’s lead citrate. Stained sections were examined using a FEI Morgagni 268 electron microscope.

### Hydrolysis of α-polyglucans with Tandem-affinity-purified ISA1–ISA2

Tandem affinity purified ISA1–ISA2 was incubated with 200 µg glucan (potato amylopectin, maize, β-limit dextrin, or oyster glycogen, all purchased from Sigma-Aldrich), in 50 mL Tris-HCL, pH 7.2, 5% (v/v) glycerol, 5 mM DTT, 1 mM CaCl_2_, 1 mM MgCl_2_ at 30°C. At different time points, aliquots were withdrawn and 0.1 M NaOH was added to stop the reaction. Samples were clarified by centrifugation (16,000 g, 5 min, 20°C) prior to high-performance anion-exchange chromatography on a Dionex Carbopac PA-200 column with pulsed anion amperometric detection (HPAEC-PAD). Gradient conditions were as described previously [10].

### Recombinant Expression of ISA1 and ISA2 in *E. coli*

The ISA1 and ISA2 cDNAs without their transit peptide sequences were obtained with gratitude from Professor Alan Myers (Iowa State University, Ames, Iowa, USA). The cDNAs were transferred into pDONR221 by Gateway recombinant cloning (Invitrogen), according to the supplier’s instructions. The cDNAs were cloned into Gateway-compatible expression vector and introduced into chemically competent DH5α or BL21 cells by heat-shock transformation. Positive clones were selected on solid LB medium containing the appropriate antibiotics. Co-expression of the following vector combinations was performed in BL21 cells; active ISA = P0GWA-ISA1 and pET29a-ISA2 or pET29a-ISA1 and P0GWA-ISA2; inactive ISA = P0GWA-ISAD367A and pET29a-ISA2. Primers for the creation of the D367A mutant of ISA1 were as follows: forward 5′-CAT GTT GAC GGC TTC CGC TTT GCT CTT GGT TCA ATC ATG-3′, reverse 5′-CAT GAT TGA ACC AAG AGC AAA GCG GAA GCC GTC AAC ATG-3′. The altered base is underlined.

### Extraction and Analysis of Glucans from *E. coli*

*E. coli* strains were grown overnight at 37°C in LB medium supplemented with the appropriate antibiotics. The culture was diluted 1∶100 in Kornberg medium (63 mM K_2_HPO_4_, 62 mM KH_2_PO_4_, 3% (w/v) yeast extract, 2% [w/v) glucose) supplemented with antibiotics and grown at 37°C until an OD_600_ of 0.5–0.6 was reached. Protein expression was induced with 1 mM IPTG and the cultures were transferred to 22°C for 20 h. To induce glycogen production, the cells were pelleted at 3000 g for 15 min, washed once with M9 medium (48 mM Na_2_HPO_4_, 22 mM KH_2_PO_4_, 9 mM NaCl, 19 mM NH_4_Cl, 2 mM MgCl_2_m 100 mM CaCl_2_, 2% (w/v) glucose), resuspended in M9 medium and incubated at 37°C for 4 h. Cells were harvested by centrifugation as described above and washed twice with 2% (w/v) mannitol. The cell pellet was frozen in liquid nitrogen and stored at −80°C.

Unless otherwise noted, all steps of glucan extraction were performed at 4°C. The cell pellet was resuspended in water, homogenised three times in an M-110P microfluidiser (Microfluidics, Newton, MA, USA) and immediately quenched with perchloric acid (final concentration of 1.12 M). The homogenate was fractionated into insoluble and soluble fractions by centrifugation at 3000 g for 10 min. The soluble fraction (the supernatant) was neutralised to pH 5–6 with 2 M KOH, 0.4 M MES, 0.4 M KCl, and the potassium perchlorate precipitate was removed by centrifugation as before. Soluble glucans in the supernatant were precipitated at −20°C in 80% methanol, pelleted by centrifugation at 16000 g for 10 min, washed with 75% methanol, dried and resuspended in water. Non-precipiatable glucans were recovered by drying the first methanol supernatant in a vacuum concentrator and resuspending the pellet in water. The insoluble fraction was washed several times with water until a neutral pH was reached and finally resuspended in water.

Quantification of glucans from the insoluble fraction, the precipitated soluble fraction and the supernatant from precipitation was done as previously described [34]. For ultra-filtration of glucans, neutralised soluble fraction was loaded onto Vivaspin 2 centrifugal concentrators with PES membrane and a 50 kDa cut-off pore size and spun with 4000 g for 10 to 15 min. The filtrate was collected, freeze dried and resuspended in water. The retentate was obtained by washing the filter 3 times with water and resuspended the retained glucans in water.

Iodine absorption spectra of glucans were obtained in CaCl_2_ solution as described [36], but with only the half amount of iodine-iodide solution in the iodine reagent. The absorption spectrum was measured on a microtitreplate reader (Infinite M1000, Tecan). Chain length distributions were obtained on a Dionex Carbopac PA-200 column with pulsed anion amperometric detection (HPAEC-PAD) as described in Streb et al. [19].

## Supporting Information

Figure S1 **Multiple sequence alignment of the plant isoamylases, ISA1 and ISA2, with isoamylase from** ***Pseudomonas amyloderamosa*** **.** UniProt identifiers or GenBank accessions for the sequences: *P. amyloderamosa* ISO CAA00929.1; Arabidopsis AtISA1 O04196; AtISA2 Q8L735; Garden pea (*Pisum sativum*) PsISA1 Q105A2; PsISA2 Q105A1; Maize (*Zea mays*) ZmISA1 O22637; ZmISA2 Q84UE6; Rice (*Oryza sativa*) OsISA1 O80403; OsISA2 gi:51038091; Potato (*Solanum tuberosum*) StISA1 Q84YG7; StISA2 Q84YG6. The eight residues absolutely conserved in all active members of the α-amylase superfamily are indicated with black arrows.
(TIF)

Figure S2 **Transmission electron micrographs of** ***E. coli*** **cells expressing the active (top) or inactive (bottom) forms of the ISA1–ISA2 isoamylase.**
(TIF)

Table S1 **Proteins identified after tandem affinity purification of** ***ISA2-TAP*** **and wild-type extracts.**
(DOCX)

Dataset S1 **Protein identification in gel slices after tandem affinity purification of ISA2-TAP protein.**
(XLSX)
