# Supplementary figures and images for: The Heteromultimeric Debranching Enzyme Involved in Starch Synthesis in Arabidopsis Requires Both Isoamylase1 and Isoamylase2 Subunits for Complex Stability and Activity
Source: PLoS One. 2013 Sep 30;8(9):e75223. doi: 10.1371/journal.pone.0075223 (PMC3787081; doi:10.1371/journal.pone.0075223)

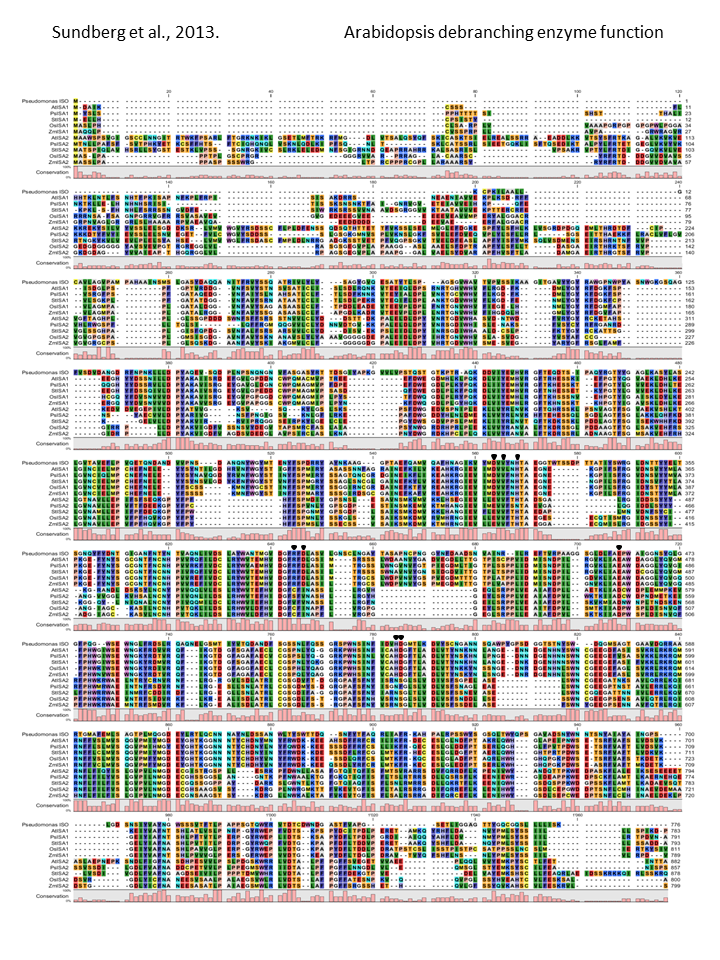

Supplement: Figure S1 — Multiple sequence alignment of the plant isoamylases, ISA1 and ISA2, with isoamylase from Pseudomonas amyloderamosa . UniProt identifiers or GenBank accessions for the sequences: P. amyloderamosa ISO CAA00929.1; Arabidopsis AtISA1 O04196; AtISA2 Q8L735; Garden pea (Pisum sativum) PsISA1 Q105A2; PsISA2 Q105A1; Maize (Zea mays) ZmISA1 O22637; ZmISA2 Q84UE6; Rice (Oryza sativa) OsISA1 O80403; OsISA2 gi:51038091; Potato (Solanum tuberosum) StISA1 Q84YG7; StISA2 Q84YG6. The eight residues absolutely conserved in all active members of the α-amylase superfamily are indicated with black arrows. (TIF) [file pone.0075223.s001.tif]
