# Supplementary material for: The Heteromultimeric Debranching Enzyme Involved in Starch Synthesis in Arabidopsis Requires Both Isoamylase1 and Isoamylase2 Subunits for Complex Stability and Activity
Source: PLoS One. 2013 Sep 30;8(9):e75223. doi: 10.1371/journal.pone.0075223 (PMC3787081; doi:10.1371/journal.pone.0075223)
Supplement: Table S1 — Proteins identified after tandem affinity purification of ISA2-TAP and wild-type extracts. (DOCX) [file pone.0075223.s003.docx]

**Supplementary Table 1: Proteins identified after tandem affinity purification of ISA2-TAP and wild-type extracts.**

Proteins from *ISA2-TAP* or wild type plants were subjected to the tandem affinity purification protocol, SDS-PAGE, in-gel tryptic digests and the peptides identified by LC-MS/MS.
